# Beyond chronological age: frailty, vitality, and subjective health structured by psychological segmentation in older adults

**DOI:** 10.3389/fragi.2026.1850676

**Published:** 2026-06-11

**Authors:** Eline Jasmijn Mertens, Damien S. E. Broekharst, Nathascha Hanzen, Karla de Rooij, Michel van Agthoven, Sjaak Bloem

**Affiliations:** 1 Marketing & Supply Chain Management, Nyenrode Business Universiteit, Breukelen, Netherlands; 2 Janssen-Cilag, Breda, Netherlands

**Keywords:** aging, frailty, older adults, psychological segmentation, subjective health experience, vitality

## Abstract

**Background:**

Chronological age remains a dominant factor in clinical decision-making despite its limited ability to reflect individual differences in functioning, vulnerability, and care needs. Age-based decision-making may contribute to exclusion from care and reinforce ageism. Psychological segmentation models, such as the Subjective Health Experience (SHE) model, classify individuals based on acceptance and perceived control of their health condition and have been proposed as a more individualized alternative. However, the relationship between SHE segments and established geriatric assessments such as frailty has not been examined.

**Objective:**

This study examined how frailty, subjective health experience, and vitality relate to psychological profiles defined by the SHE model, and whether chronological age adds explanatory value beyond segmentation.

**Methods:**

A cross-sectional questionnaire study was conducted among community-dwelling Dutch adults aged ≥67 years (N = 753). Frailty was assessed using the Groningen Frailty Indicator (GFI), subjective health via a self-anchored ladder scale, and vitality via the Vita-16 instrument. Participants were classified into four SHE segments based on mean scores of ≥5.0 per dimension on acceptance and perceived control. Nonparametric analyses were used, with additional age-stratified comparisons (67–75 vs. ≥76 years).

**Results:**

Frailty, subjective health, and vitality varied significantly across segments (all p < 0.001). Frailty prevalence ranged from 30% in segment 1%–74% in segment 4 (Cramer’s V = 0.41), with low acceptance emerging as the strongest differentiating factor. Segment 1 (high acceptance/high control) showed the most favorable outcomes; segment 4 (low acceptance/low control) showed the poorest. Frailty distinguished intermediate segments more clearly than vitality, with segment 3 (low acceptance/high control) showing higher frailty prevalence than segment 2 (69% vs. 40%). Chronological age did not differ across segments and did not moderate the association between segmentation and health outcomes, except for limited age effects on vitality in segments 1, 2, and 4.

**Conclusion:**

Psychological segmentation via the SHE model explains meaningful variation in frailty, subjective health, and vitality in older adults, independent of chronological age. Frailty assessment combined with SHE segmentation enables a transition from age-based to needs-based care, supporting more tailored support strategies for older adults.

## Introduction

Global life expectancy has steadily increased in recent decades, making the concept of healthy aging a central focus in geriatric healthcare practice and research (Campisi et al., 2019; World Health Organization, 2024) denotes healthy aging as preserving the functional abilities that enable wellbeing in older individuals (Beard et al., 2016; World Health Organization, 2024). This paradigm emphasizes not only the absence of disease but also the preservation of physical, mental, and social health.

Despite this multidimensional view of aging, chronological age remains a dominant factor in care decisions. The use of age-based decision-making has led to the systematic exclusion of older adults from treatments such as surgery, a practice widely criticized as a form of ageism (Butler, 1969; Chang et al., 2020; Levy, 2017; Romero-Ortuno and O’Shea, 2013). Meta-analyses and reviews have addressed the adverse health outcomes resulting from ageism, ranging from psychosocial impacts to poorer physical health (Chang et al., 2020; Lamont et al., 2015; Meisner, 2012). Additionally, chronological age is a limited predictor of functional status, resilience, and healthcare outcomes (Dent et al., 2019; São et al., 2019; Theou and Rockwood, 2012). These limitations have led healthcare professionals to urge a transition from age-based to needs-based models of care, underpinned by individualized assessments of physical and functional capacity and related needs (Chang et al., 2020; Clegg et al., 2013; Westerhof et al., 2014).

Geriatric assessments such as frailty, subjective health and vitality instruments have gained increasing attention in this context (Toba et al., 2002; Peel et al., 2004; Schuurmans et al., 2004; Bloem, 2008). Frailty is defined as a state of heightened vulnerability arising from age-related decline across physiological systems, significantly impairing the ability to cope with daily or acute stressors (Clegg et al., 2013; Xue, 2011; Doody et al., 2023; Dent et al., 2023). It is closely associated with an increased risk of hospitalization, emphasizing the importance of early detection (Dent et al., 2019). The subjective health experience delineates an individual’s experience of physical and mental functioning while living their life in the way they want to within the actual constraints and limitations of individual existence (Bloem, 2008; Bloem et al., 2020). The concept of vitality pertains to an individual’s capacity and intrinsic drive to autonomously uphold a mode of existence conducive to living, growing, and developing in a vigorous, dynamic, and spirited manner (Toba et al., 2002; Broekharst et al., 2023; Solberg et al., 2012; Strijk et al., 2015).

While standardized assessments, such as the Groningen Frailty Indicator (GFI), help identify individuals at risk, they focus primarily on physical and functional deficits. These tools offer limited insight into how older adults perceive and cope with their health status and provide little guidance on how to tailor support accordingly (Dent et al., 2019; Westerhof et al., 2014). For example, older adults with similar frailty scores may differ significantly in their subjective experience of health and support strategies; these differences are not captured by such assessments alone.

**FIGURE 1:**
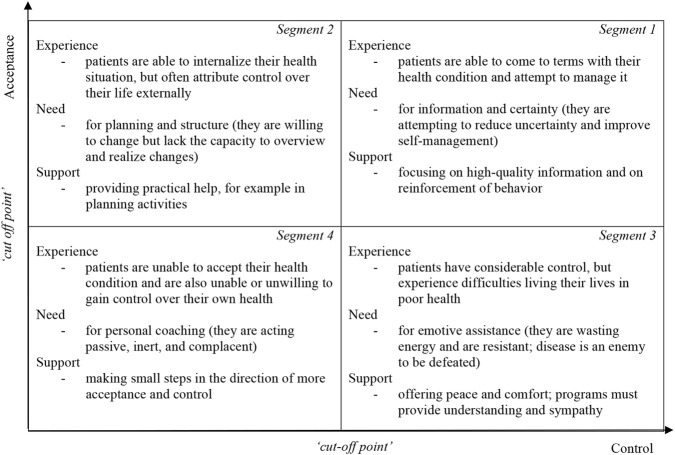
The Subjective Health Experience model.

Segmentation models have been developed to address how individuals interpret and manage their health condition. A widely leveraged segmentation model in healthcare is the Subjective Health Experience (SHE) model developed by Bloem et al. (2020), Broekharst et al. (2022) (Figure 1). This segmentation model classifies adults into four segments on the basis of the two psychological determinants of subjective health: acceptance and control. Each segment is associated with distinct support strategies, ranging from informational support to intensive personal coaching tailored specifically to the psychological needs of the individual (Figure 1, (Bloem et al., 2020; Broekharst et al., 2022; Broekharst et al., 2025)).

Given the wide range of available geriatric assessments, it is critical to evaluate which instruments, or combinations thereof, offer the most meaningful insights for tailoring support. Whereas instruments measuring frailty or vitality predominantly assess *what* individuals experience in terms of functioning, segmentation models such as the SHE model address *how* individuals perceive and adapt to their health status.

In contrast to conventional health measures, the SHE model identifies actionable psychological profiles, that are defined by acceptance and perceived control. These profiles directly inform which type of support is most appropriate for a given individual.

While prior research has linked vitality and subjective health to SHE segments, (Broekharst et al., 2023), frailty has not yet been examined in this context. First, by exploring how frailty, vitality, and subjective health relate to the psychological profiles of the SHE model, this study aims to further refine the characterization of the four segments. Second, we assess whether chronological age contributes additional explanatory value by comparing age-related variation in each assessment across segments. Finally, we examine whether and which of these assessments can be meaningfully combined to enhance individualized support strategies. These findings may assist healthcare professionals in offering more personalized and effective support, ultimately contributing to the promotion of healthy aging.

## Methods

### Study design and participants

This cross-sectional observational study used data from an online questionnaire distributed to a panel of community-dwelling older adults in the Netherlands. The participants were members of the Ouderenfonds (National Foundation for Older Adults) research panel and were invited via email to complete the questionnaire. Data collection occurred in February and March 2021. Eligible participants were aged 67 years or older. All the respondents provided informed consent for their responses to be used for research purposes.

### Data collection

The following data were extracted from the database: demographic data (age, sex, education level, household size, living arrangement, and urbanization), validated instruments assessing frailty (GFI), subjective health experience, vitality (Vita-16), and segmentation via the Subjective Health Experience (SHE) model. These demographic variables were included to descriptively characterize the study population and provide contextual information relevant to subjective health, frailty, and functioning in older adults. The GFI is a validated self-report measure suitable for community-based survey research (Schuurmans et al., 2004). Unlike performance-based instruments such as the Fried frailty phenotype (Fried et al., 2001) or clinician-rated scales such as the Clinical Frailty Scale (Rockwood et al., 2005), it does not require objective measurement or trained assessors, making it the most appropriate instrument for the present study design.

## Assessments

### Frailty

Frailty was measured via the GFI, an instrument covering three dimensions: “daily activities”, “psychosocial functioning” and “health problems” (Schuurmans et al., 2004; Van Damme et al., 2021). The GFI consists of 15 items dichotomized for scoring. The total frailty score was calculated by summing the number of affirmative responses, yielding a score between zero (not frail) and 15 (very frail). A cutoff score of ≥4 was applied to identify participants as frail, in line with previous studies (Andela et al., 2010; Baitar et al., 2013).

### Subjective health experience: ladder measure

Subjective health experience, a dimension of health-related quality of life, was assessed via an 11-point self-anchored visual ladder scale. The participants were asked to evaluate their health experience with the following question: “How would you rate your experienced health this past month, compared to the worst and best day you had during that month?” The scale ranged from 0 (worst day) to 10 (best day) (Bloem, 2008; Broekharst et al., 2023).

### Subjective health experience: segmentation

The participants were segmented into four groups on the basis of their responses to six items assessing acceptance (three items) and perceived control (three items) (Figure 1) (Broekharst et al., 2022). The questions regarding acceptance were as follows: (1) “I am at peace with my health condition”, (2) “The way in which I am functioning physically and mentally is acceptable to me”, and (3) “I accept my health condition the way it is”. The three questions about perceived control were as follows: (1) “I have the feeling that I have grip on my health condition”; (2) “My health condition is to a great extent in my own power”; and (3) “I have a lot of influence on my health condition.” All the items were rated on a 7-point Likert scale ranging from 1 (fully disagree) to 7 (fully agree). On the basis of previous work by Bloem et al. (2020), mean scores of ≥5.0 per dimension were used to classify participants as high on that dimension, distinguishing four segments (Bloem et al., 2020). This yielded four distinct segments: (1) high acceptance/high control, (2) high acceptance/low control, (3) low acceptance/high control, and (4) low acceptance/low control.

### Vitality

Vitality was assessed via the Vita-16 questionnaire, which evaluates three core dimensions—energy (five items), motivation (six items), and resilience (five items)—and provides both subscale scores and an overall vitality score. All the items were rated on a 7-point Likert scale ranging from 1 (seldom) to 7 (always). To ensure comparability, item scores were averaged for each dimension. The overall vitality score was then calculated via a weighted formula: ((0.4 × energy) + (0.3 × motivation) + (0.3 × resilience), as applied in a previous study (Broekharst et al., 2023).

### Data analysis

Descriptive summaries are reported as percentages for categorical variables and as medians with interquartile ranges (median ± IQR) for continuous variables. The internal consistency of the instruments was assessed via Cronbach’s alpha, with α > 0.70 considered acceptable (Bernstein and Nunally, 1994).

The Shapiro‒Wilk test was used to assess normality; as assumptions were not met, nonparametric tests were performed. Differences across the four segments were analyzed via the Kruskal‒Wallis test. Significant overall results were subsequently explored through pairwise comparisons between segments by applying Bonferroni corrections, which were chosen for their strict and robust control for multiple testing (Bland and Altman, 1995). Chi-square tests were used to analyze categorical variables (e.g., frailty status), with Cramér’s V as the effect size.

Prior to age stratification, complementary analyses of covariance (ANCOVAs) were conducted to examine whether the associations between SHE segmentation and frailty, vitality, and subjective health remained after controlling for chronological age. Age was entered as a covariate and SHE segment as the between-subjects factor. Although the primary analyses were non-parametric because normality assumptions were not fully met, ANCOVAs were included as complementary analyses given the large sample size and the robustness of general linear models to moderate deviations from normality. These analyses were performed to assess whether the observed segment differences persisted after controlling for age.

To further explore potential age-related patterns within segments, age-stratified analyses were subsequently conducted. Participants were divided into two age groups: 67–75 years and ≥76 years. Mann‒Whitney U tests were used to compare age groups within each segment. Owing to the insufficient sample size in segment 3 for the older group (n = 4), this segment was excluded from the age-stratified analyses. No merging of segments was applied; comparisons were therefore conducted across segments 1, 2, and 4 only. Continuous variables derived from Likert scales (subjective health experience and vitality) were normalized to a 0–1 scale to facilitate comparisons across measures with different original score ranges, where 0 represented the lowest possible value and 1 represented the highest possible value on the original scale. The data were analyzed via IBM SPSS Statistics (version 29.0; IBM Corp., Armonk, NY, United States). Figures were generated via MATLAB R2024b (MathWorks, Natick, MA, United States) and prepared for publication in Adobe Illustrator 2024 (Adobe Inc., San Jose, CA, United States). A significance level of *p* < 0.05 was applied.

## Results

### Participant characteristics

Data were collected from 753 adults aged 67 years or older (median age = 74 years, range 67–100). Participant characteristics are listed in Table 1 and largely resembled the Dutch older population, except for a higher rate of independent living (98.9%) (CBS. Males and females, 2022; CBS. Bevolking, 2021; CBS, 2023).

**TABLE 1:** Participant characteristics.

Characteristic	Category	%
Age	Median (IQR)	74 (70–79) years
	Range	67–100 years
Gender	Male	40.6%
	Female	59.4%
Education level	Primary education	3.5%
	Secondary education	47.7%
	Higher education	38.6%
	University education	10.3%
Household size	1 person	51.3%
	2 persons	47.3%
	3 persons or more	1.5%
Living arrangement	Independent	98.9%
	Not independent	1.1%
Urbanization	Urban	58.0%
	Rural	42.0%

### Scale reliability

The subjective health assessment included three questions on acceptance (Cronbach’s α = 0.92) and control (α = 0.88). Vitality, including energy (α = 0.94), motivation (α = 0.95), and resilience (α = 0.92), was assessed via the Vita-16 scale (α = 0.96). Frailty was measured via the Groningen frailty indicator (GFI) (α = 0.73). All scales showed acceptable reliability, exceeding the Cronbach’s alpha coefficient threshold (α > 0.70).

### Frailty and segmentation

GFI scores were compared to examine whether frailty levels differed across segments. This revealed a significant difference in frailty levels across segments (χ^2^(3) = 179.71, *p* < 0.001). Segment comparisons revealed that participants in segments 3 and 4 had significantly higher GFI scores than those in segments 1 (both *p* < 0.001) and 2 (*p* = 0.004 and *p* < 0.001, respectively). No significant differences were found between segments 1 and 2 or between segments 3 and 4.

**FIGURE 2:**
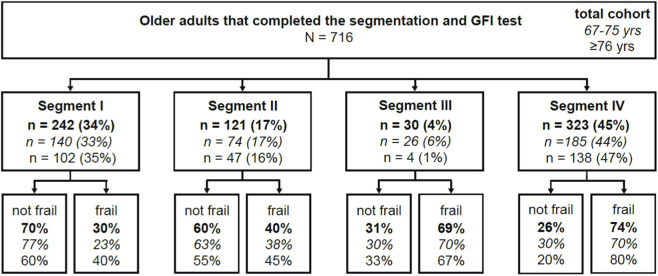
Distribution of older adults across segments and frailty status. Distributions are shown for the total cohort (bold), adults aged 67–75 years (italics), and adults aged 76 years and older (regular font). The segment proportions were horizontally accumulated to represent the entire cohort. Within each segment, the cohort was subdivided into frail and nonfrail groups.

On the basis of the GFI, participants were classified as “frail” (GFI ≥4) or “not frail.” Frailty prevalence varied significantly across the segments (χ^2^(3) = 112.80, *p* < 0.001, Cramer’s V = 0.41). The highest prevalence of frailty was observed in segments 4 (74%) and 3 (69%) compared with segments 2 (40%) and 1 (30%) (Figure 2; Table 2). These findings suggest that low acceptance (segments three and 4) is more strongly associated with frailty than is low perceived control (segments 2 and 4).

**TABLE 2:** Summary of outcome measures per segment.

Variable	Segment 1 Median [IQR]	Segment 2 Median [IQR]	Segment 3 Median [IQR]	Segment 4 Median [IQR]
GFI-score	2.0 [1.0–4.0]	3.0 [2.0–4.0]	5.0 [3.0–6.0]	6.0 [3.0–7.0]
% Frail	30%	40%	69%	74%
Subjective health	8.0 [7.0–8.0]	7.5 [7.0–8.0]	7.0 [5.8–7.3]	6.0 [5.0–7.0]
Vitality total	5.5 [4.7–6.0]	4.5 [3.8–5.5]	4.4 [3.9–5.0]	3.4 [2.6–4.4]
Energy	5.4 [4.6–6.2]	4.4 [3.4–5.6]	4.0 [3.1–4.6]	3.0 [2.0–4.2]
Motivation	5.3 [4.5–6.0]	4.5 [3.5–5.3]	4.3 [3.7–5.1]	3.7 [2.3–4.5]
Resilience	5.4 [5.0–6.0]	5.0 [4.2–5.6]	5.2 [4.4–5.6]	4.0 [3.0–5.0]

Data are presented as medians with interquartile ranges. Frailty was expressed as the percentage of frail individuals (GFI ≥4).

To examine whether differences in frailty between segments remained significant after controlling for chronological age, an ANCOVA was conducted with age entered as a covariate and SHE segment as the between-subjects factor. After controlling for age, SHE segment remained significantly associated with frailty (segments: F(3,661) = 86.49, p < 0.001). Chronological age was also significantly associated with frailty, although the effect was substantially smaller (age: F(1,661) = 30.16, p < 0.001).

To further explore the distribution of age across the segments and support the age-stratified analyses, we additionally examined whether chronological age varied across the segments. Age did not significantly differ across the four segments in the full sample or within either age group (67–75 and ≥76 years). Similarly, pairwise comparisons revealed no significant age differences between individual segments. These findings suggest a relatively comparable age distribution across segments.

Next, we determined whether frailty prevalence differed across age groups (67–75 years and >75 years) and found that it was significantly lower in the younger group than in the older group in segment 1 (*χ* ^*2*^(1) = 6.86, *p* = 0.009, Cramer’s V = 0.17). Given the limited sample sizes within each age group for segment 4, age-stratified frailty comparisons for this segment should be interpreted with caution.

### Subjective health experience and segmentation

Subjective health experience partly contributes to the segmentation model, therefor analyses involving subjective health were intended primarily to further characterize the four segments.

To examine differences in subjective health experiences in the past month, ladder scores were compared across segments, revealing a significant difference across segments (χ^2^(3) = 199.27, *p* < 0.001). Segment 1 reported significantly higher scores than segments 3 and 4 did (both *p* < 0.001), but did not differ significantly from segment 2. Segment 2 scored significantly higher than segments 3 (*p* = 0.006) and 4 (*p* < 0.001). No significant differences were observed between segments 3 and 4. Table 2 provides an overview of the outcomes.

ANCOVA revealed that, after controlling for chronological age, SHE segment remained significantly associated with subjective health experience (segments: F(3,711) = 84.47, p < 0.001), whereas chronological age itself was not significantly associated with subjective health experience (age: F(1,711) = 2.90, p = 0.089). To further explore potential age-related patterns within segments, age-stratified analyses were subsequently conducted. No significant differences were found between the age groups within the segments. These findings indicate that segmentation explains variations in subjective health experiences more effectively than age does.

### Vitality and segmentation

Next, we investigated whether vitality varied across segments. Significant differences were found in energy (χ^2^(3) = 259.52, *p* < 0.001), motivation (χ^2^(3) = 157.69, *p* < 0.001), resilience (χ^2^(3) = 174.69, *p* < 0.001), and total vitality (χ^2^(3) = 253.86, *p* < 0.001). For all the domains, segment 4 consistently reported the lowest vitality scores, whereas segment 1 reported the highest scores.

Given that the total vitality score encompasses all vitality domains, pairwise comparisons were conducted to further investigate differences across segments. Compared with all the other segments, segment 1 presented significantly greater total vitality. Segment 2 exhibited lower vitality than did segment 1 but higher vitality than did segment 4 (*p* < 0.001), reflecting intermediate vitality levels. Segment 4 had the lowest vitality scores, which were lower than those of segments 1 and 2 (*p* < 0.001) and 3 (*p* = 0.01). No significant differences were observed between segments 2 and 3. This suggests a general trend of segment 1 > segment 2 ≈ segment 3 > segment 4.

ANCOVA revealed that, after controlling for chronological age, SHE segment remained significantly associated with vitality (segments: F(3,691) = 133.95, p < 0.001). Chronological age was also significantly associated with vitality, although the effect was substantially smaller (age: F(1,691) = 31.51, p < 0.001).

To further explore potential age-related patterns within segments, differences in age groups (67–75 vs. ≥76 years) were subsequently examined within each segment. Energy levels varied between the younger and older age groups in segments 2 (*U* = 1015.0, *p* < 0.001) and 4 (*U* = 9888.5, *p* = 0.01). Motivation varied between age groups in segments 1 (*U* = 5645.5, *p* = 0.03), 2 (*U* = 1251.0, *p* = 0.02), and 4 (*U* = 9500.0, *p* = 0.002). Resilience did not vary across age groups in segments 1 and 2 but varied across age groups in segment 4 (*U* = 9867.0, *p* = 0.009). The total vitality varied between age groups in segments 1 (*U* = 5675.0, *p* = 0.04), 2 (*U* = 1201.5, *p* = 0.007), and 4 (*U* = 9535.0, *p* = 0.002). An overview of the age group outcomes is shown in Table 3.

**TABLE 3:** Summary of outcome measures per segment for the total cohort and the younger and older age groups.

Variables	Segment 1: total cohort Median [IQR]	*Segment 1 (67–75) Median [IQR]*	Segment 1 (>75) Median [IQR]	Segment 2: total cohort Median [IQR]	*Segment 2 (67–75) Median [IQR]*	Segment 2 (>75) Median [IQR]	Segment 3: total cohort Median [IQR]	*Segment 3 (67–75) Median [IQR]*	* Segment 3 (>75)	Segment 4: total cohort Median [IQR]	*Segment 4 (67–75) Median [IQR]*	Segment 4 (>75) Median [IQR]
GFI-score	**2.0 [1.0–4.0]**	*2.0 [1.0–3.0]*	3.0 [1.0–4.0]	**3.0 [2.0–4.0]**	*3.0 [1.3–4.0]*	3.0 [2.0–5.0]	**5.0 [3.0–6.0]**	*5.0 [3.0–6.0]*	​	**6.0 [3.0–7.0]**	*5.0 [3.0–7.0]*	6.0 [4.0–8.0]
% Frail	**30%**	*23%*	40%	**40%**	*38%*	45%	**69%**	*70%*	​	**74%**	*70%*	80%
Subjective health experience	**8.0 [7.0–8.0]**	*8.0 [7.0–8.0]*	8.0 [7.0–8.0]	**7.5 [7.0–8.0]**	*8.0 [7.0–8.0]*	7.0 [7.0–8.0]	**7.0 [5.8–7.3]**	*7.0 [5.0–7.0]*	​	**6.0 [5.0–7.0]**	*6.0 [5.0–7.0]*	6.0 [5.0–7.0]
Vitality Total	**5.5 [4.7–6.0]**	*5.6 [4.9–6.1]*	5.3 [4.4–5.8]	**4.5 [3.8–5.5]**	*4.7 [4.0–5.6]*	4.1 [3.7–4.9]	**4.4 [3.9–5.0]**	*4.3 [3.7–4.9]*	​	**3.4 [2.6–4.4]**	*3.7 [2.7–4.6]*	3.2 [2.5–3.9]
Vitality Energy	**5.4 [4.6–6.2]**	*5.6 [4.8–6.2]*	5.2 [4.0–6.2]	**4.4 [3.4–5.6]**	*5.0 [4.0–6.0]*	4.0 [3.0–4.8]	**4.0 [3.1–4.6]**	*3.9 [3.0–4.4]*	​	**3.0 [2.0–4.2]**	*3.3 [2.2–4.4]*	2.8 [2.0–3.8]
Vitality Motivation	**5.3 [4.5–6.0]**	*5.5 [4.7–6.2]*	5.2 [4.2–5.7]	**4.5 [3.5–5.3]**	*4.8 [3.7–5.5]*	3.8 [2.8–5.2]	**4.3 [3.7–5.1]**	*4.2 [3.7–4.8]*	​	**3.7 [2.3–4.5]**	*3.8 [2.3–4.8]*	3.2 [2.2–4.2]
Vitality Resilience	**5.4 [5.0–6.0]**	*5.6 [5.0–6.0]*	5.4 [5.0–5.8]	**5.0 [4.2–5.6]**	*5.0 [4.0–5.6]*	4.9 [4.2–5.6]	**5.2 [4.4–5.6]**	*5.2 [4.1–5.6]*	​	**4.0 [3.0–5.0]**	*4.0 [3.0–5.2]*	3.8 [2.8–4.6]

Total cohort (bold), adults aged 67 -75 years (italics), and adults aged 76 years and older (regular font). Bold indicates summary total cohort, whereas italics indicate the younger age group and regular font the 76 years and older age group. Data are presented as medians with interquartile ranges. Frailty was expressed as the percentage of frail individuals (GFI ≥4). *Segment 3 was excluded from the age-stratified analysis owing to the small sample size.

### Frailty, subjective health and vitality across segments

**FIGURE 3:**
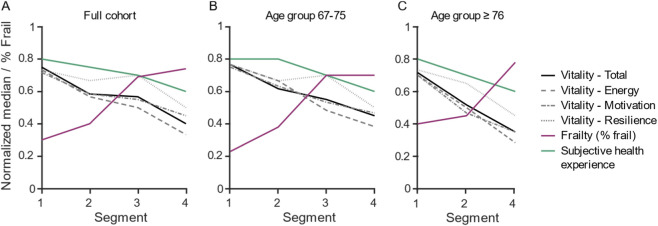
Trends in normalized vitality, subjective health experiences, and frailty prevalence across the four segments. **(A)** Trends are shown across the four segments for the full cohort. Segment 1 reflects high acceptance and high control; segment 2, high acceptance and low control; segment 3, low acceptance and high control; and segment 4, low acceptance and low control. The proportion of frail participants in each segment is shown. **(B)** Same as **(A)** but stratified for the age group of 67–75 years. **(C)** Same as **(A)** but stratified by the age group of 76 years and older.

To provide an integrated overview of the assessment outcomes, Figure 3 presents the distributions of frailty prevalence, subjective health experiences, and vitality across the four segments. These trends were largely consistent across the two age groups (67–75 years and ≥76 years), suggesting that segmentation explains the variation in frailty, subjective health, and vitality more effectively than chronological age does.

## Discussion

In this study, we characterized the four psychological segments of the Subjective Health Experience (SHE) model using frailty, subjective health experience, and vitality. We investigated how these assessments varied across segments and examined their susceptibility to age-related differences (Bloem et al., 2020; Broekharst et al., 2023). To enable tailored support, it is important to consider both objective indicators of functioning and the subjective dimension of health experience. Frailty identifies who is functionally vulnerable, but does not capture how individuals experience, interpret, and respond to their limitations. The SHE model addresses precisely this subjective dimension, enabling a transition from risk identification to needs-based, tailored support. By comparing these assessments across segments, we refined the interpretation of each psychological profile. We then evaluated the extent to which outcomes were shaped by chronological age. Finally, we considered how combining these instruments may improve the tailoring of support strategies to individual needs.

First, our study characterized how frailty, vitality, and subjective health experience varied across the segments. Segment 1 (high acceptance and control) was associated with the highest vitality and subjective health scores, which is consistent with previous findings (Broekharst et al., 2023). This study extends the characterization of segment 1 by demonstrating that it also has the lowest level of frailty. This observation aligns with recent longitudinal research indicating that greater psychological resilience is associated with slower frailty progression over time (Ye et al., 2024). In contrast, segment 4 (low acceptance and control) reported the least favorable outcomes across all domains. Together, these findings reflect a clear gradient in health status across the four segments, from most to least favorable. A closer comparison between intermediate segments provides further insights: segment 2 (high acceptance, low control) demonstrated better subjective health and lower frailty than did segment 3 (low acceptance, high control), emphasizing the crucial role of acceptance in perceived health outcomes, which is consistent with previous research (Van Erp et al., 2023; Stalpers, 2009). Our results support the view that, even in the absence of perceived control, older adults who have come to terms with health limitations are more closely linked to wellbeing.

The subjective health experience, assessed via the ladder scale, varied consistently across segments and was aligned with expectations, reinforcing the internal consistency of the SHE model. Because subjective health experience partly contributes to the segmentation model itself, these findings should primarily be interpreted as further characterization of the segment profiles rather than as evidence of a fully independent construct. The ladder was not significantly associated with chronological age, making it a stable cross-check indicator. Unusually high ladder scores in psychologically vulnerable segments could indicate mismatches needing clinical attention, although this was not observed in our data (Bloem, 2008).

Interestingly, despite lower frailty and higher subjective health in segment 2, vitality levels did not differ significantly from those in segment 3. This suggests that vitality does not fully reflect the psychological mechanisms that distinguish how individuals navigate their health. Nevertheless, vitality broadly mirrored the segment gradient and offered insight into the energetic, resilience and motivational dimensions of wellbeing. In this way, it enriched the characterization of the psychological profiles by adding experiential nuance to the perceived energy and driving their health status.

Second, we examined how age affected the various assessments by comparing age groups. This was particularly relevant given the widespread critique of chronological age as a basis for healthcare decisions, which has contributed to ageist practices and the systematic exclusion of older adults from certain treatments (Chang et al., 2020; Levy, 2017; Romero-Ortuno and O’Shea, 2013). Vitality consistently varied with age, with older adults (76+ years) reporting lower scores than younger adults (67–75 years). In contexts where psychological segmentation aims to move beyond chronological age as the primary organizing principle, relying on vitality may unintentionally reintroduce age-based reasoning. This age sensitivity may limit the usefulness of support strategies intended to be inclusive across older populations and may inadvertently perpetuate age-based reasoning in care decisions (Chang et al., 2020; São et al., 2019).

Frailty was less influenced by age, which aligns with previous research suggesting that frailty, when properly assessed, offers a more reliable basis for identifying vulnerability than age alone (Dent et al., 2019; Clegg et al., 2013; Doody et al., 2023). Age-related differences in frailty were observed only in segment 1, where younger individuals (67–75 years) presented exceptionally low frailty levels, the lowest across all segments and age groups, indicating that this was not part of a broader or consistent age trend but rather a segment-specific outlier. Additionally, frailty showed stronger alignment with segment profiles. Segments 2 and 3, for example, were clearly distinguished in frailty, with the latter showing a higher prevalence. While frailty and vitality both reflect aspects of functioning and wellbeing, our findings suggest that they capture overlapping but not identical constructs.

Thus, given its greater segment alignment and relative age stability, frailty may offer a more robust and inclusive foundation for tailoring support across diverse older populations. However, while frailty reflects observable limitations in functioning (the *what*), it does not capture the subjective dimension: *how* individuals interpret, cope with, and respond to their health status. Frailty assessment can serve as an initial tool to identify vulnerability in older adults, but segmentation via the SHE model is needed to understand their needs and tailor support accordingly. This combined approach enables professionals to move from identifying risk to delivering segment-specific support. This distinction is evident in our findings, as one in three individuals in segment 1, typically characterized by high acceptance and control, was classified as frail but reported high levels of vitality and subjective health. These individuals appear to maintain functional engagement despite their frailty status, in line with previous research (Nyende et al., 2023). This perspective is further supported by Shimizu et al. (2023), who reported that frail older adults with high subjective health maintained social participation levels comparable to those of nonfrail individuals. These cases illustrate the added value of combining frailty assessment with psychological segmentation. While frailty identifies vulnerability, the SHE model sheds light on how individuals experience and navigate those limitations, and what type of support best fits their needs (Figure 1). This combination allows for more targeted support. For example, a psychologically resilient but frail person in segment 1 may need only reassurance and information, whereas a similarly frail individual with low control in segment 2 could benefit more from structured planning and practical help. Detailed segment-specific strategies are available in Broekharst et al. (2023), and the SHE model has recently been applied to predict subjective health experience in older cancer survivors.

This study has several strengths, including the use of a large community-based sample and validated instruments for assessing frailty, vitality, and segmentation. This is also the first study to explicitly examine frailty via the SHE model. However, this study had several limitations. First, the cross-sectional design precludes causal inference; the observed associations between psychological segmentation and frailty, vitality, and subjective health should be interpreted as correlational. Second, the sample included only independently living older adults in the Netherlands, limiting the generalizability of the findings to institutionalized populations or other cultural contexts. Panel participants may represent a relatively engaged population. Third, the small sample size in segment 3 among the oldest participants prevented stratified age analyses for this group. Fourth, because the GFI includes psychosocial components and the segmentation model captures psychological aspects of health experience, some conceptual overlap between these constructs should be considered when interpreting the observed associations. Finally, the overlapping distributions between adjacent segments indicate substantial intra-individual variation, which may limit the precision of individual-level stratification. The SHE model is therefore best applied at the care-pathway level rather than as a single deterministic classification tool. These findings point to several directions for future research. First, longitudinal studies are needed to examine whether psychological segmentation predicts frailty progression over time, and whether targeted segment-specific interventions can slow this progression. Second, although gender distribution did not significantly differ across segments, future studies may explore potential gender effects on the relationship between psychological segmentation and health outcomes. Third, validation of the combined SHE-frailty approach in institutionalized or clinically frail populations would extend the generalizability of the present findings. Finally, implementation studies are needed to evaluate the feasibility and effectiveness of integrating psychological segmentation into routine geriatric assessments in clinical practice.

## Conclusion

This study demonstrated that frailty, vitality, and subjective health experience are meaningfully structured by the Subjective Health Experience (SHE) model. Frailty aligned most clearly with psychological profiles and remained relatively stable across age, making it a robust indicator of functional limitations. Subjective health, as measured by the ladder, also consistently reflected segment differences and was not significantly associated with chronological age, reinforcing its value as a supportive, cross-check indicator. Vitality offered insights into the characterization of the segments but was more sensitive to age and less effective in distinguishing between segments 2 and 3, limiting its utility in age-inclusive care.

By integrating frailty assessment with psychological segmentation, we move beyond identifying vulnerability to understanding how older adults perceive, adapt to, and manage their health limitations. This combined perspective supports more tailored support that reflects both objective functioning and subjective experience, which is consistent with calls for holistic approaches to aging and care planning (Theou and Rockwood, 2012; Westerhof et al., 2014; Van Damme et al., 2021).

## Data Availability

The datasets presented in this article are not readily available because the dataset contains responses from older adults collected via an existing research panel. Data are anonymized and available upon reasonable request to the corresponding author. Data cannot be shared publicly to protect the privacy of panel participants in accordance with applicable Dutch privacy legislation (AVG/GDPR). Requests to access the datasets should be directed to e.mertens@nyenrode.nl.
